# Vascularization of a Bone Organoid Using Dental Pulp Stem Cells

**DOI:** 10.1155/2023/5367887

**Published:** 2023-05-09

**Authors:** Aonan Li, Jun-Ichi Sasaki, Gabriela L. Abe, Chihiro Katata, Hirohiko Sakai, Satoshi Imazato

**Affiliations:** ^1^Department of Dental Biomaterials, Osaka University Graduate School of Dentistry, Osaka, Japan; ^2^Department of Advanced Functional Materials Science, Osaka University Graduate School of Dentistry, Osaka, Japan; ^3^Department of Restorative Dentistry and Endodontology, Osaka University Graduate School of Dentistry, Osaka, Japan

## Abstract

Bone organoids offer a novel path for the reconstruction and repair of bone defects. We previously fabricated scaffold-free bone organoids using cell constructs comprising only bone marrow-derived mesenchymal stem cells (BMSCs). However, the cells in the millimetre-scale constructs were likely to undergo necrosis because of difficult oxygen diffusion and nutrient delivery. Dental pulp stem cells (DPSCs) are capable of differentiating into vascular endothelial lineages and have great vasculogenic potential under endothelial induction. Therefore, we hypothesized that DPSCs can serve as a vascular source to improve the survival of the BMSCs within the bone organoid. In this study, the DPSCs had greater sprouting ability, and the proangiogenic marker expressions were significantly greater than those of BMSCs. DPSCs were incorporated into the BMSC constructs at various ratios (5%–20%), and their internal structures and vasculogenic and osteogenic characteristics were investigated after endothelial differentiation. As a result, the DPSCs are differentiated into the CD31-positive endothelial lineage in the cell constructs. The incorporation of DPSCs significantly suppressed cell necrosis and improved the viability of the cell constructs. In addition, lumen-like structures were visualized by fluorescently labelled nanoparticles in the DPSC-incorporated cell constructs. The vascularized BMSC constructs were successfully fabricated using the vasculogenic ability of the DPSCs. Next, osteogenic induction was initiated in the vascularized BMSC/DPSC constructs. Compared with only BMSCs, constructs with DPSCs had increased mineralized deposition and a hollow structure. Overall, this study demonstrated that vascularized scaffold-free bone organoids were successfully fabricated by incorporating DPSCs into BMSC constructs, and the biomimetic biomaterial is promising for bone regenerative medicine and drug development.

## 1. Introduction

Bone regeneration and reconstruction remain a challenge because critical-size defects are not spontaneously healed by the body [1, 2]. Because of the limitations associated with conventional grafting materials, tissue engineering is considered a promising approach for bone regenerative medicine [3, 4]. Bone organoids, based on tissue engineering, are three-dimensional (3D) structures derived from stem cells, progenitor cells, or differentiated cells that self-organize through cell-cell and cell-matrix interactions to recapitulate aspects of the native bone architecture and function in vitro [5, 6]. Bone organoids are a prospective biomaterial for drug screening and bone regeneration, and several techniques, including scaffolds, have been developed to prepare bone organoids [7–9].

We previously demonstrated that scaffold-free bone organoids could be fabricated from bone marrow-derived mesenchymal stem cells (BMSCs) using a 3D cell construct technique with temperature-responsive hydrogels [10, 11]. The BMSC constructs have a self-organizing capacity and form bone matrices via the endochondral ossification process [10]. However, the cells in the millimetre-scale construct were likely to undergo necrosis because of the lack of oxygen and nutrient delivery, which highlighted the significance of vascularization inside the bone organoid [12]. Therefore, it is essential that adequate vascularization within the organoid supports the nutrient supply and guarantees cell survival in vitro, which further facilitates anastomosis of the organoid to host vasculature after transplantation in vivo [13, 14]. To date, numerous approaches have been attempted to develop vascularization inside biomimetic tissues, including bone organoids [15, 16].

A prevascular network was formed by mixing BMSCs and human umbilical vein endothelial cells (HUVECs) in hydrogel scaffolds [17]. The study also demonstrated synergistic interactions between the BMSCs and HUVECs that upregulate osteogenic differentiation of the cell/hydrogel composites. Similarly, our group incorporated HUVECs into the scaffold-free BMSC constructs and observed that capillary-like structures were formed inside the constructs with the deposition of bone matrices [18]. Despite the promise of these approaches coculturing BMSCs with endothelial cells, there are concerns on the application of endothelial cells or their progenitor cells in a clinical setting. Specifically, host tissues are damaged to harvest endothelial cell lineages, and further processes are required for cell purification [19, 20]. Although these cells can be obtained, it is difficult to acquire sufficient cell numbers for tissue regeneration [21]. Therefore, exploring an alternative to endothelial cells is necessary for further progress in bone organoid engineering.

Dental pulp stem cells (DPSCs) are categorized as mesenchymal stem cells that have high performance in cell proliferation and multilineage differentiation, and they are used in the field of tissue engineering and dental pulp regeneration [22–24]. DPSCs constantly express the receptor of vascular endothelial growth factor (VEGF) and have the ability to differentiate into the endothelial cell lineage [25, 26]. Additionally, capillary vessels developed by DPSCs are able to anastomose with the host vasculature when a DPSC-loaded scaffold was implanted in a mouse model [27–29]. These characteristics make DPSCs a prospective source of endothelial cells to form the vascular network in tissue organoids.

In this study, we hypothesized that DPSCs can display an endothelial differentiation ability and form a capillary-like structure within the BMSC constructs. To achieve the vascularization of BMSC constructs, DPSCs should have greater differentiation ability toward endothelial cells than BMSCs. The purpose of this study was to investigate the endothelial differentiation ability of BMSCs and DPSCs and further fabricate a vascularized bone organoid by incorporation of DPSCs into BMSC constructs.

## 2. Materials and Methods

### 2.1. Cell Culture

Human BMSCs derived from iliac crests (Lonza, Basel, Switzerland) and DPSCs isolated from adult third molars (Lonza) were cultured in growth medium (GM) comprising Dulbecco's modified Eagle's medium (DMEM; Wako, Osaka, Japan) supplemented with 20% fetal bovine serum (Thermo Fisher Scientific, MA, USA) and 1% penicillin-streptomycin (Sigma-Aldrich, MO, USA). Green fluorescent protein- (GFP-) labelled DPSCs were used to track their localization inside cell constructs. DPSCs at passages 3–5 were transduced with GFP using a lentivirus vector (Sigma-Aldrich) and selected by 1 *μ*g/mL puromycin (Sigma-Aldrich) for at least 1 week.

To induce the endothelial differentiation, each cell type and cell constructs were treated with an endothelial differentiation medium (EM; EGM2-MV (Lonza) supplemented with 50 ng/mL rhVEGF_165_ (R&D Systems, MN, USA)). For osteogenic differentiation, cell constructs were maintained in osteogenic differentiation medium (OM) comprising GM supplemented with dexamethasone (1 × 10^−6^ mol/L; Sigma-Aldrich), *β*-glycerophosphate disodium salt hydrate (1 × 10^−2^ mol/L; Sigma-Aldrich), ascorbic acid (50 *μ*g/mL; Sigma-Aldrich), and calcium chloride (1 × 10^−2^ mol/L; Wako). Cells and cell constructs were incubated in a humidified incubator at 37°C with 5% CO_2_.

### 2.2. Capillary Sprouting Assay

BMSCs and DPSCs were seeded onto 24-well plates coated with Growth Factor-Reduced Matrigel (Corning, NY, USA) at a density of 1 × 10^4^ cells/well and cultured with EM for 1, 3, 7, and 14 days. Micrographs of the reticular structure formed by the cells were captured with a light microscope (TE2000; Nikon, Tokyo, Japan) equipped with a digital camera. The number of branches and their lengths were measured using ImageJ software (National Institutes of Health, MD, USA) (*n* = 4).

### 2.3. Real-Time Reverse Transcription Polymerase Chain Reaction (RT-PCR)

BMSCs and DPSCs at an initial density of 500 cells/cm^2^ were seeded on the culture dish and maintained in EM. After 1, 3, 7, and 14 days, mRNA expressions of *vascular endothelial growth factor A* (*VEGFA*), *C-X-C motif chemokine ligand 1* (*CXCL1*), *Nanog*, and *glyceraldehyde 3-phosphate dehydrogenase* (*GAPDH*) (Thermo Fisher Scientific) were evaluated using a Cells-to-Ct Kit (Thermo Fisher Scientific) (*n* = 4). The expressions of *VEGFA*, *CXCL1*, and *Nanog* relative to *GAPDH* were calculated using the 2^−*ΔΔ*Ct^ method. One of the replicates from the BMSCs group with one-day induction was utilized to normalize all samples.

### 2.4. Fabrication of the BMSC/DPSC Constructs

The spherical cell constructs were fabricated according to a previous method using a temperature-responsive poly-N-isopropylacrylamide (pNIPAAm) hydrogel as a temporary support for cell aggregation [11]. Briefly, a curable resin mould with hemispherical domes (*φ* = 1.5 mm) was designed using graphic modelling software (Freeform; 3D Systems, SC, USA) and manufactured with a 3D printing system (Eden; Stratasys, Rehovot, Israel). The polymerized hydrogel with semispherical sockets was formed using a resin mould and then used for the preparation of cell constructs. Cell suspensions with different ratios of BMSCs to DPSCs (BMSCs/DPSCs = 100/0, 95/5, 90/10, 80/20) were seeded onto the sockets of the pNIPAAm gel and cultured at 37°C for 24 h. BMSC constructs and BMSC/DPSC constructs were then harvested from the hydrogel by cooling at room temperature for 15 min and then transferring to 60 mm^2^ culture dishes. Consequently, each cell construct had approximately 5 × 10^5^ cells in total. These cell constructs were then cultured with EM for up to 20 days on a seesaw shaker to prevent the cells from adhering. The size change of the constructs was recorded using a CCD camera (DS-Fi2; Nikon) equipped with a stereoscopic microscope (SMZ745T; Nikon) (*n* = 5). Cell constructs comprising only BMSCs and those containing 20% DPSCs were referred to as 100B/0D and 80B/20D, respectively.

### 2.5. Live/Dead Staining

The viability of the cells comprising the cell construct was evaluated at 5, 10, and 20 days of culture. Specimens were washed with phosphate-buffered saline (PBS; Thermo Fisher Scientific) and then stained with calcein AM and ethidium homodimer 1 (Thermo Fisher Scientific) for living and dead cells, respectively. The constructs were cut in half using a surgical scalpel, mounted on glass slides, and observed under a fluorescence microscope (TE2000) at a 475 nm of wavelength for live cells and 559 nm for dead cells.

### 2.6. Histological Evaluation of the Vascularized BMSC/DPSC Constructs

Cell constructs were cultured in EM and harvested at 5, 10, and 20 days. Hematoxylin-eosin (HE) staining was conducted on 5 *μ*m thick sections, and the stained sections were observed by a CCD camera (DS-Fi2) mounted on a light microscope (ECLIPSE Ci-L, Nikon).

Immunofluorescence staining was carried out to investigate the localization of endothelial differentiated cells, which were positive for CD31. Sections were deparaffinized, rehydrated with a sequence of phased dilutions of ethanol, and subjected to epitope retrieval with trypsin (Sigma-Aldrich). After blocking with PBS containing 0.3% Triton X-100 (Alfa Aesar, MA, USA) and 0.1% bovine serum albumin (Sigma-Aldrich), sections were incubated with a mouse monoclonal anti-human CD31 antibody (Agilent Technologies, CA, USA) and a rabbit polyclonal antibody against GFP (Thermo Fisher Scientific). These first antibodies were visualized using Alexa Fluor 488 goat anti-mouse IgG and Alexa Fluor 594 goat anti-rabbit IgG (Thermo Fisher Scientific). Cell nuclei were stained with Hoechst33342 (Thermo Fisher Scientific), and then, sections were observed by a fluorescence microscope (TE2000).

To observe the lumen-like structures formed inside cell organoids, fluorescence imaging was also conducted using fluorescein isothiocyanate- (FITC-) labelled nanoparticles. Cell constructs with endothelial differentiation up to 20 days were immersed in PBS containing FITC-dextran beads (150 kDa; Sigma-Aldrich) for 2 h. The constructs were washed, cut in half with a scalpel, and observed under a fluorescence microscope at a wavelength of 490 nm (TE2000).

### 2.7. Osteogenic Differentiation of the Vascularized BMSC/DPSC Constructs

The cell constructs were cultured with EM for 20 days, followed by culture in OM for another 20 days. Mineralized matrices in the tissue sections were investigated by von Kossa staining. Sections were deparaffinized and washed with distilled water before staining with a 5% silver nitrate solution (Sigma-Aldrich) for 5 min under UV light irradiation. Stained sections were then immersed in a 5% sodium thiosulfate solution (Wako) for 5 min, and a light microscope (ECLIPSE Ci-L) was used to observe mineralized deposition within cell constructs. Furthermore, to determine whether the vessel-like structure was maintained after osteogenic induction, the FITC-dextran bead technique described above was performed on vascularized constructs.

### 2.8. Statistical Analysis

One-way analysis of variance with Tukey's honestly significant difference test was used for comparison of more than two groups. A Student's *t*-test was used for comparison of two groups. *p* < 0.05 was considered a significant difference.

## 3. Results

### 3.1. Comparison of the Vasculogenic Potential of BMSCs and DPSCs

**Figure 1:**
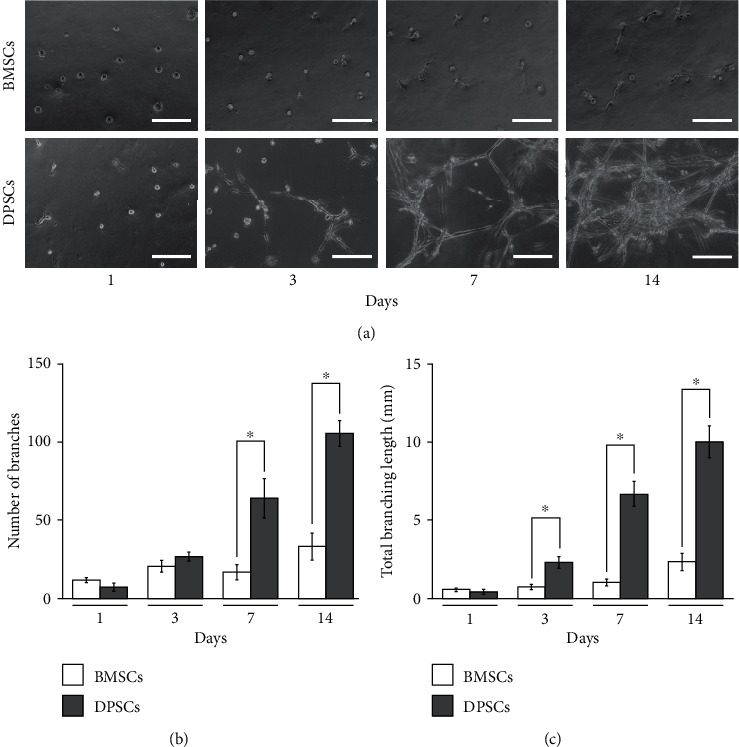
Comparison of the sprouting ability of BMSCs and DPSCs. (a) Photomicrographs of sprouting capillary structures formed by BMSCs and DPSCs with endothelial induction. (b) Quantitative analyses of the number of branches and (c) total branching length at corresponding time points. Scale bars: 200 *μ*m. ^∗^*p* < 0.05; mean ± SD; *n* = 4.

DPSCs started to sprout in the first 3 days of endothelial induction, and a reticular-like structure was gradually formed for up to 14 days (Figure 1(a)). Despite the presence of a few sprouts in BMSCs at 7 days, cell sprouts were unable to connect to each other to develop capillary-like networks, even after 14 days of induction. Image analysis revealed that both the number and total length of branches formed by DPSCs were significantly greater than those formed by BMSCs throughout the experimental period (Figures 1(b) and 1(c)).

**Figure 2:**
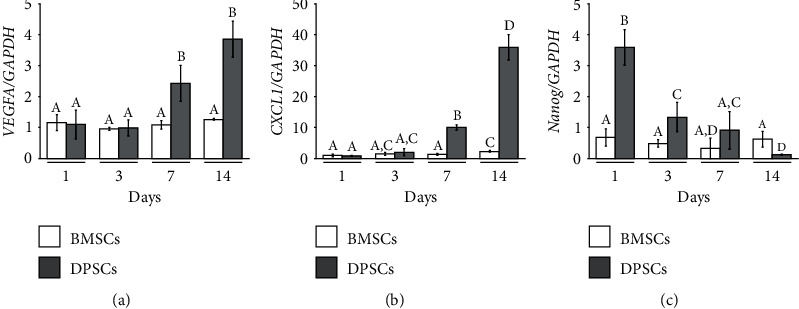
Comparison of endothelial differentiation ability of BMSCs and DPSCs. mRNA expression of (a) *VEGFA*, (b) *CXCL1*, and (c) *Nanog* after 1, 3, 7, and 14 days of endothelial induction. Different capital letters indicate significant differences among groups (*p* < 0.05); mean ± SD; *n* = 4.

The endothelial differentiation ability of BMSCs and DPSCs was evaluated by real-time PCR (Figure 2). The gene expressions of proangiogenic markers *VEGFA* and *CXCL1* were upregulated in DPSCs after 7 days of induction, whereas no difference in *VEGFA* expression was observed in the BMSCs. Although a slight increase in *CXCL1* expression was observed in the BMSCs at 14 days, the fold change was significantly less than that of the DPSCs, specifically 2.24 ± 0.24 and 35.81 ± 4.92 for the BMSCs and DPSCs, respectively. Conversely, the expression of the stemness marker *Nanog* was incrementally downregulated in DPSCs while invariable in BMSCs over the culture period.

### 3.2. Endothelial Differentiation of the BMSC/DPSC Constructs

**Figure 3:**
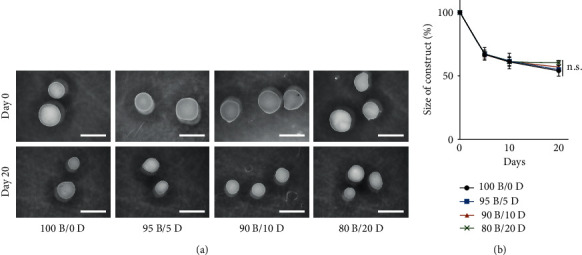
Morphological alteration of BMSC/DPSC constructs after endothelial differentiation. (a) Stereoscopic images and (b) size variation of BMSC/DPSC (100B/0D–80B/20D) constructs up to 20 days of culture. The initial size of the cell construct (day 0) was defined as 100%. Scale bars: 1 mm. n.s.: not significant; mean ± SD; *n* = 3.

Stereoscopic images and size alterations of BMSC/DPSC constructs are presented in Figure 3. The spherical morphology was maintained in all specimens over the culture period, and no significant difference in size was observed at the indicated time points. The size of the cell construct decreased to approximately 60% among all groups by 10 days of culture, and less size change was observed thereafter.

**Figure 4:**
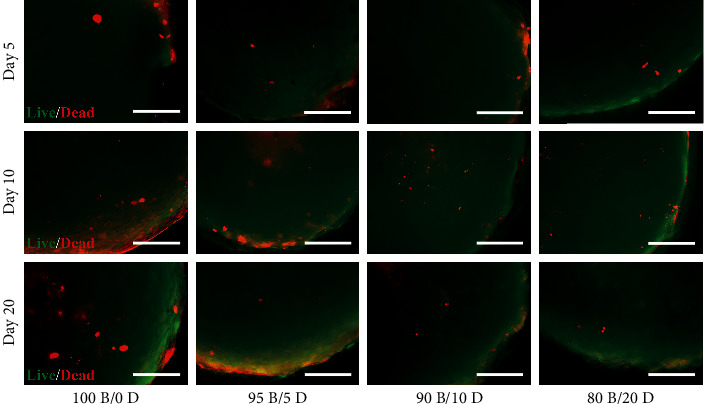
Cell viability within the BMSC/DPSC constructs under endothelial induction. Scale bars: 50 *μ*m.

Live/dead staining revealed that the BMSC/DPSC constructs mainly comprise living cells at day 5 (Figure 4). At longer culture times, dead cells were distributed throughout the constructs comprising solely of BMSCs (100B/0D), and the incorporation of DPSCs reduced the number of dead cells. Notably, there were few dead cells in the cell constructs containing 20% DPSCs (80B/20D) throughout the culture period.

**Figure 5:**
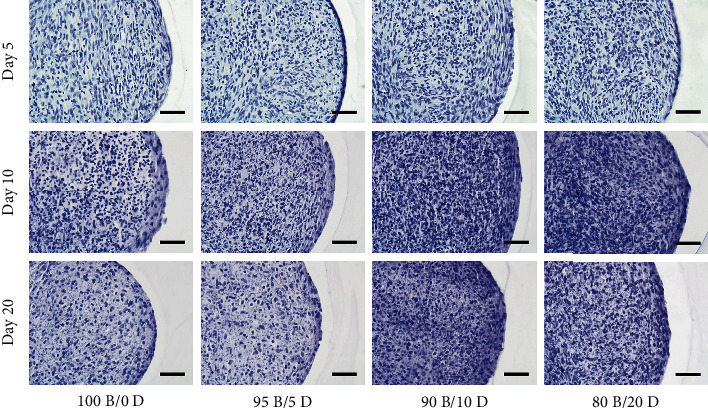
HE staining of BMSC/DPSC constructs after 5, 10, and 20 days of endothelial differentiation. Scale bars: 50 *μ*m.

Images of the HE staining indicated that all specimens had 2–3 outermost cell layers and an internal uniform cellular structure (Figure 5). All specimens showed a similar structure at day 5; however, the number of stained cells then gradually increased as the DPSC ratio increased for up to 20 days.

### 3.3. Vascularized Structures of the BMSC/DPSC Constructs

**Figure 6:**
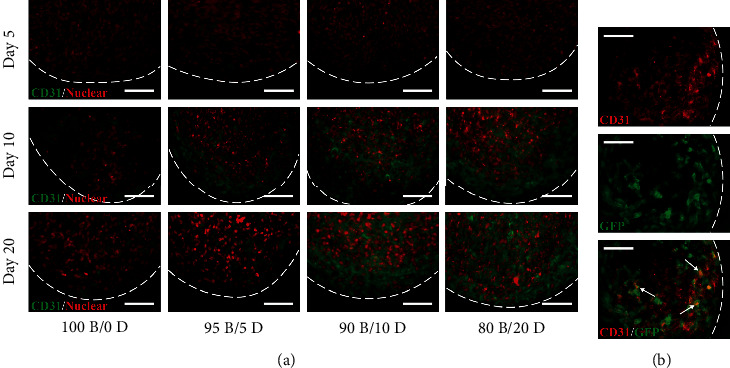
Immunofluorescence staining of BMSC/DPSC constructs after endothelial differentiation. (a) CD31 expression in BMSC/DPSC constructs at 5, 10, and 20 days. (b) Photomicrographs of CD31-positive cells (red) and GFP-labelled DPSCs (green) in 80B/20D constructs after 20 days of endothelial differentiation. White arrows indicate the colocalization of CD31 and GFP-transduced DPSCs. Dotted lines indicate the outline of cell constructs. Scale bars: 50 *μ*m.

CD31, a specific marker of endothelial cells, was rarely expressed in the specimens at day 5 (Figure 6(a)). Thereafter, CD31-positive cells gradually increased with the increasing ratio of DPSCs in the construct. Notably, CD31 was expressed throughout the 80B/20D constructs after 20 days of endothelial induction, whereas only a few CD31-positive cells were observed in the constructs with only BMSCs (100B/0D). Furthermore, GFP-transduced DPSCs expressed CD31 within the construct (Figure 6(b)).

**Figure 7:**
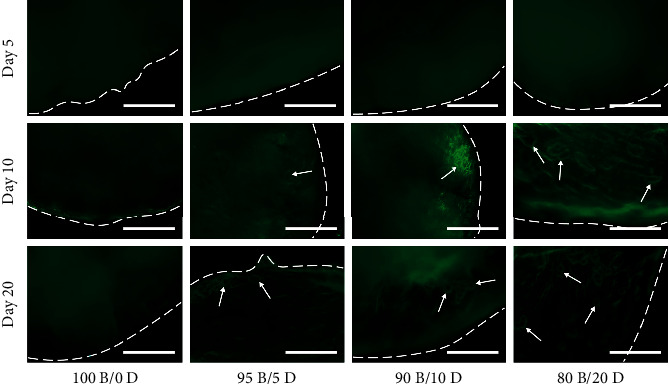
Fluorescence imaging of BMSC/DPSC constructs after 5, 10, and 20 days of endothelial induction. White arrows indicate the capillary-like hollow structures formed within the cell constructs. Dotted lines indicate the outline of the cell constructs. Scale bar: 50 *μ*m.

Fluorescence imaging revealed that all specimens showed a homogeneous structure at day 5, while a capillary-like lumen structure was observed in the DPSC-incorporated cell constructs after 10 days of culture (Figure 7). The number of vascular-like hollow structures increased during the induction period; however, no vascularized structures were observed in the cell constructs without DPSCs (100B/0D), even after 20 days of culture.

### 3.4. Structures of the Vascularized Bone-Like BMSC/DPSC Constructs

**Figure 8:**
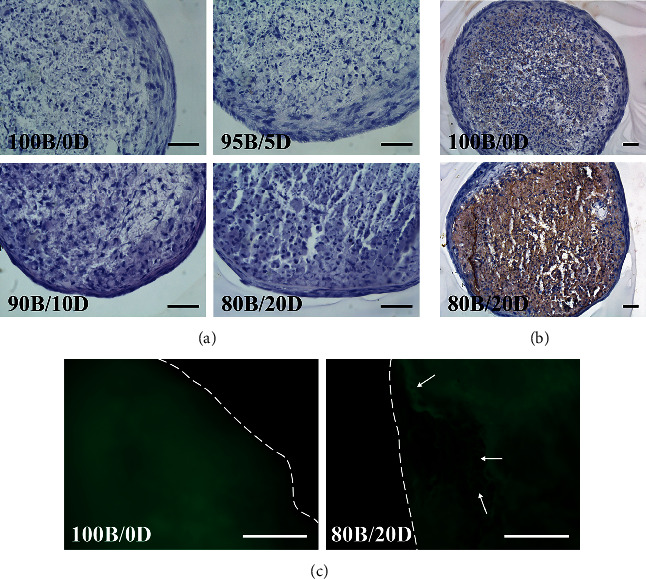
Structure of the cell constructs after endothelial and osteogenic induction. (a) HE staining, (b) von Kossa staining, and (c) fluorescence imaging of cell constructs with varying ratios of BMSCs and DPSCs. White arrows indicate a capillary-like hollow structure in the mineralized 80B/20D constructs. Dotted lines indicate the outline of the cell constructs. Scale bars: 50 *μ*m.

Histological analyses were performed on the cell constructs after 20 days of endothelial induction and 20 days of osteogenic induction. The images of HE staining showed that all cell constructs maintained their morphology and structure (Figure 8(a)). Regarding the bone matrices, the cell constructs with only BMSCs (100B/0D) formed a few mineralized nodules, while DPSC-incorporated constructs significantly increased the precipitated mineralized matrices (Figure 8(b)).

To determine whether the vascularized structures remained after osteogenic induction, fluorescence imaging was conducted for osteo-induced organoids (Figure 8(c)). Capillary-like hollow structures were visualized in the 80B/20D constructs, indicating that the vascularized structure of the cell construct was maintained during the osteogenic induction. Additionally, cell constructs comprising only BMSCs (100B/0D) showed no vascularized structure after osteogenic induction and endothelial differentiation.

## 4. Discussion

The main challenge for in vitro engineering of functional tissues is to ensure vascular formation in biomaterials, including organoids [30, 31]. In this study, we attempted to employ DPSCs instead of primary endothelial cells (e.g., HUVECs) as a vascular source for cell constructs, and the influence of DPSCs on the vasculogenic and osteogenic properties of BMSC constructs was investigated.

It was previously demonstrated that both BMSCs and DPSCs can differentiate into vascular endothelial lineages [32–34]. This study revealed that DPSCs have greater sprouting capacity because they can form significantly more branches and develop longer branching than BMSCs. Genetically, the expressions of proangiogenic mRNA (e.g., *VEGFA* and *CXCL1*) in DPSCs were significantly greater than those in BMSCs after 7 days of induction. Notably, the stemness marker of *Nanog* decreased in DPSCs while no significant difference was detected in BMSCs. These results indicated that the endothelial differentiation ability of DPSCs was significantly greater than that of BMSCs, and the stemness property was maintained in BMSCs with 14 days of endothelial induction.

Next, cell constructs comprising different ratios of BMSCs and DPSCs were fabricated using a temperature-responsive gel mould. The cell constructs formed by self-assembly of cells without an exogenous scaffold were able to mimic the extracellular microenvironment and the following activation of cell response to the external milieus [35, 36]. In this study, BMSC/DPSC constructs maintained their spherical shape during long-term culture, which was attributed to the high expression of neural- (N-) cadherin in BMSCs and vascular endothelial- (VE-) cadherin in DPSCs [27, 37]. Both molecules are highly involved in the regulation of cell-cell adhesion and allow isolated cells to assemble into cell constructs [38, 39]. These results indicated that scaffold-free cell constructs with BMSCs and DPSCs have a self-organization ability that facilitates the formation of artificial biomimetic tissues in vitro.

Improving the survival rate of cells is significant for organoids. In this study, the viability of cells in the cell construct was improved with the inclusion of DPSCs. DPSC-incorporated constructs increased the expression of CD31, and colocalization of CD31 and GFP further verified that DPSCs were able to differentiate into endothelial cells within the constructs. Dextran beads with FITC (average diameter: 85 Å) can more easily permeate loose areas than dense areas of tissues [22, 40, 41]. The FITC-labelled dextran beads were successfully used to visualize the vessel-like and capillary-like structures inside the BMSC/DPSC constructs, whereas no hollow structures were observed in the BMSC constructs without DPSCs. Overall, the vessel-like hollow structure formed by endothelial-differentiated DPSCs improved cell survival inside the spherical organoids. While this study revealed that the capillary network originated from DPSCs supported the viability of the cell construct, further investigations are required to determine whether DPSCs can differentiate into endothelial cells in a centimetre-scale organoid.

DPSC cell constructs formed matrices more mineralized than constructs comprising only BMSCs (100B/0D). Meanwhile, a vessel-like structure was observed in the BMSC/DPSC organoids after osteogenic differentiation of the vascularized constructs. This osteogenic enhancement might be attributed to the vascularized network formed by endothelial-differentiated DPSCs. The induction of vasculature enables engineered bone tissues to simulate the native structure and achieve effective metabolic exchange between tissues and the external environment [42–44]. Therefore, this physiological condition improved the survival rate of osteogenic cells and promoted bone formation in the BMSC/DPSC constructs. In addition, we previously reported that endothelial cells in a bone organoid secreted growth factors that support the survival and differentiation of osteogenic cells [18]. The results of this study suggested that differentiated DPSCs had a trophic effect and assisted BMSCs to produce bone matrices. The cell-cell interaction between DPSCs and BMSCs within the organoid should be further investigated.

## 5. Conclusions

The present study demonstrated that DPSCs have greater vasculogenic capacity than BMSCs, and they formed a vessel-like structure within 3D cell constructs of BMSCs. In addition, a vascularized structure facilitates cell survival and further deposition of bone matrices in the DPSC/BMSC constructs. Overall, we succeeded in fabricating vascularized biomimetic bone organoids using scaffold-free BMSC/DPSC constructs that are promising for bone regenerative medicine and drug development.

## Data Availability

The data used to support the findings of this study are available from the corresponding author upon request.
